# The therapeutic effect of radiotherapy combined with systemic therapy compared to radiotherapy alone in patients with simple brain metastasis after first-line treatment of limited-stage small cell lung cancer: a retrospective study

**DOI:** 10.1186/s12957-024-03372-y

**Published:** 2024-04-10

**Authors:** Xinyu Gao, Tingting Liu, Min Fan, Hongfu Sun, Shixuan Zhou, Yuxin Zhou, Haolin Zhu, Ru Zhang, Zhanyuan Li, Wei Huang

**Affiliations:** 1 School of Clinical Medicine, Weifang Medical University, Weifang, China; 2 Department of Radiation Oncology, Shandong Cancer Hospital and Institute, Shandong First Medical University, Shandong Academy of Medical Sciences, Jinan, China; 3 Department of Nuclear Medicine, Shandong Cancer Hospital and Institute, Shandong First Medical University, Shandong Academy of Medical Sciences, Jinan, China

**Keywords:** Small cell lung cancer, Brain metastases, Cranial radiotherapy, Systemic therapy

## Abstract

**Purpose:**

We aimed to compare the therapeutic effect of radiotherapy (RT) plus systemic therapy (ST) with RT alone in patients with simple brain metastasis (BM) after first-line treatment of limited-stage small cell lung cancer (LS-SCLC).

**Methods:**

The patients were treated at a single center from January 2011 to January 2022. BM only without metastases to other organs was defined as simple BM. The eligible patients were divided into RT alone (monotherapy arm) and RT plus ST (combined therapy arm). Univariate and multivariate Cox proportional hazards analyses were used to examine factors associated with increased risk of extracranial progression. After 1:1 propensity score matching analysis, two groups were compared for extracranial progression-free survival (ePFS), PFS, overall survival (OS), and intracranial PFS (iPFS).

**Results:**

133 patients were identified and 100 were analyzed (monotherapy arm: *n* = 50, combined therapy arm: *n* = 50). The ePFS of the combined therapy was significantly longer than that of the monotherapy, with a median ePFS of 13.2 months (95% CI, 6.6–19.8) in combined therapy and 8.2 months (95% CI, 5.7–10.7) in monotherapy (*P* = 0.04). There were no statistically significant differences in PFS (*P* = 0.057), OS (*P* = 0.309), or iPFS (*P* = 0.448). Multifactorial analysis showed that combined therapy was independently associated with better ePFS compared with monotherapy (HR = 0.617, *P* = 0.034); more than 5 BMs were associated with worse ePFS compared with 1–5 BMs (HR = 1.808, *P* = 0.012).

**Conclusions:**

Compared with RT alone, combined therapy improves ePFS in patients with simple BM after first-line treatment of LS-SCLC. Combined therapy and 1–5 BMs reduce the risk of extracranial recurrence.

## Introduction

Small cell lung cancer (SCLC) is a highly aggressive malignant tumor, accounting for approximately 15% of all lung cancers [1]. Although most SCLC patients have high sensitivity to initial chemotherapy (CT) and radiotherapy (RT), most patients relapse after first-line treatment [2, 3]. About 10% of patients have brain metastasis (BM) at the initial diagnosis, and over 50% of patients have BM during the course of the disease [4]. For limited-stage SCLC (LS-SCLC), the development of BM is a sign of systemic failure of tumor control, with survival of < 3 months for untreated SCLC BM patients compared to 3.7–8.5 months for treated SCLC BM patients [5–7]. Cranial RT is used as the primary treatment in most BM patients with SCLC [8–10]. Although cranial RT has satisfactory local control and neurological symptom relief, however, most BM patients die from systemic disease rather than intracranial failure [11]. Therefore, many physicians advocate combining systemic therapy (ST) with RT [12]. However, in patients with SCLC BM, there is no evidence-based medicine to prove whether ST combined with RT results in better survival outcomes. In order to better determine the optimal treatment plan for SCLC BM, it is necessary to choose patients with simple BM after first-line treatment of LS-SCLC, to avoid interference from ST due to initial treatment or progression of extracranial tumors. The number of patients who meet these standards is very small, we recruited 133 patients with simple BM after first-line treatment of LS-SCLC, that was, patients who only had BM with no other organ metastasis. Based on propensity score matching (PSM) analysis, we conducted a retrospective analysis using real data to evaluate whether RT combined with ST has a beneficial effect on the survival of patients with SCLC BM as compared with RT alone.

## Methods

### Study cohort

This study included 133 SCLC patients who treated at Shandong Cancer Hospital and Institute (Jinan, Shandong, China) from January 2011 to January 2022. They were patients who developed simple BM after first-line treatment failure in LS-SCLC. The inclusion criteria were as follows: (1) LS-SCLC confirmed by histology or cytology at the time of initial diagnosis; (2) previously treated with only one chemotherapy-containing treatment line; (3) BM detected by magnetic resonance imaging (MRI) or computed tomography. The exclusion criteria were: (1) history of other malignant tumors; (2) meningeal metastases; (3) incomplete demographic, clinical, and/or outcome data. Prior to initial treatment, all patients underwent contrast-enhanced MRI to evaluate the brain. This study has been approved by the Ethics Committee of Shandong Cancer Hospital and Institute (approval No. SDTHEC2023011003). All procedures involving patients were in accordance with the principles outlined in the Declaration of Helsinki.

The following information was collected from eligible patients: age at diagnosis, gender, Karnofsky Performance Status (KPS) score at diagnosis, whether prophylactic cranial irradiation (PCI) treatment was performed, and number of BMs. In addition, time to first intracranial progression and time to first extracranial progression after BM treatment were collected. Time from SCLC diagnosis to the diagnosis of BM were collected. Intracranial progression was distinguished from adverse radiological reactions (pseudo progression) according to the Neuro-Oncology BM Response Assessment Criteria [13].

Patients were reviewed at 3-month intervals for the first 2 years after initial treatment, then at 6-month intervals after 2 years of treatment, and then annually after 5 years of treatment, with prompt follow-up for any changes in condition during this period. The review included a contrast-enhanced Computed Tomography scan of the chest and upper abdomen, a contrast-enhanced MRI of the head, and a bone scan if necessary. BM were confirmed by contrast-enhanced MRI at the initial diagnosis or during the progression of the disease.

### Outcomes measured

The eligible patients were divided into RT alone (monotherapy arm) and RT combined with ST (combined therapy arm). This study compared the survival data of the two cohorts. The primary endpoint of this study was defined as extracranial progression-free survival (ePFS), which refers to the time from BM diagnosis to extracranial progression or death. Secondary endpoints included overall survival (OS), progression-free survival (PFS), and intracranial progression-free survival (iPFS). OS was defined as the time interval from BM diagnosis to death from any cause or last known follow-up. PFS was defined as the time interval from BM diagnosis to any progression, death from any cause, or last known follow-up. iPFS was defined as the time interval from BM diagnosis to interval of intracranial progression, death from any cause, or last known follow-up. Brain metastases free survival (BMFS) was defined as the time interval from SCLC diagnosis to BM diagnosis. Adverse events (AEs) were evaluated and graded based on the National Cancer Institute Common Terminology Criteria for Adverse Events (CTCAE) version 5.0.

### Statistical analysis

We compared the characteristics of the two groups of patients, with normally distributed continuous data expressed as mean ± standard deviation and categorical variables expressed as percentages. Statistical differences between groups were analyzed using the Chi-square test or Fisher’s exact test for categorical variables and the unpaired t-test or one-way analysis of variance (ANOVA) for continuous variables. Survival curves were constructed using the Kaplan-Meier method, and Log-rank tests were used to compare differences in survival curves. Hazard ratios (HR) were calculated using univariate and multivariate Cox proportional risk models to examine factors associated with extracranial progression. This study analyzed the impact of BMFS on prognosis, defined the median of BMFS as the optimal cutoff value. Use univariate Cox proportional risk models to compare whether there was a statistical difference in the impact of BMFS on iPFS, ePFS, PFS, and OS. We used SPSS software version 26.0 (IBM-SPSS Inc, Armonk, NY) for all statistical analyses. All tests were two-sided, and a *P* value of < 0.05 was considered statistically significant.

Propensity scores were generated using multivariate logistic regression modeling, PSM was used to reduce the difference in baseline variables between the monotherapy and combined therapy. PSM includes age, gender, KPS at diagnosis, whether PCI treatment was performed, and the number of BMs. On the basis of PSM, patients were matched using the nearest neighbor method with a 1:1 pairwise ratio and a caliper size value of 0.02.

## Results

### Baseline characteristics

According to the inclusion and exclusion criteria, a total of 133 patients were finally enrolled in this study, grouped according to the treatment modality after BM: 67 patients in the monotherapy and 66 patients in the combined therapy. Between the two groups in the original dataset, the number of patients who underwent PCI in the combined therapy was 14 (21.2%), which was more than that in the monotherapy, which was 6 (9%) (*P* = 0.048). Age, gender, KPS at diagnosis, and number of BMs were evenly distributed between the two groups. After 1:1 PSM, the differences between groups were equal, yielding matched cohorts of 50 patients each. As shown in Table 1, the covariates were well balanced with no significant differences between groups.

**Table 1:** Baseline characteristics of patients before and after matching

Characteristic		Before matching				After matching	
	Monotherapy (*n* = 67)	Combined therapy (*n* = 66)	*P* value		Monotherapy (*n* = 50)	Combined therapy (*n* = 50)	*P* value
Age, years	58.9 ± 8.5	59.4 ± 10.6	0.800		58.8 ± 9.1	60.2 ± 10.6	0.473
Gender			0.875				0.817
Female	16(23.9)	15(22.7)			12(24)	13(26)	
Male	51(76.1)	51(77.3)			38(76)	37(74)	
KPS score			0.411				0.826
90–100	50(74.6)	45(68.2)			36(72)	35(70)	
70–80	17(25.4)	21(31.8)			14(28)	15(30)	
PCI			0.048				1.000
Yes	6(9.0)	14(21.2)			6(12)	6(12)	
No	61(91.0)	52(78.8)			44(88)	44(88)	
Number of brain metastasis			0.094				1.000
1–5	33(49.3)	42(63.6)			30(60)	30(60)	
> 5	34(50.7)	24(36.4)			20(40)	20(40)	

**Abbreviations:** *KPS =* karnofsky performance status; *PCI =* prophylactic cranial irradiation

### Treatment protocol

The initial treatment plan for all patients was synchronous RT and CT. The CT regimen was etoposide combined with platinum, and the RT regimen included conventional fractionation (60–66 Gy in 30–33 once-daily fractions) and hyperfractionation RT (45 Gy in 30 twice-daily fractions).

Table 2 shows specific BM treatment plans for patients after PSM. RT included whole brain radiotherapy (WBRT) (20–40 Gy in 10–20 fractions), WBRT combine stereotactic radiosurgery (SRS) (30 Gy in 10 fractions or 40 Gy in 20 fractions for whole brain with additional 10–15 Gy for BM) and SRS (30–45 Gy in 10–15 fractions). ST contained CT and CT plus immune checkpoint inhibitors (ICIs), median treatment cycle were 4 cycles. There were no significant intergroup differences in the RT modalities (*P* = 0.643).

**Table 2:** Specific BM treatment plans for patients

	Monotherapy (*n* = 50)	Combined therapy (*n* = 50)	*P* value
Radiotherapy			0.643
SRS	5(10)	8(16)	
WBRT	20(40)	20(40)	
WBRT + SRS	25(50)	22(44)	
Systemic therapy			
CT		40(80)	
CT + ICIs		10(20)	

**Abbreviations:***SRS =* stereotactic radiosurgery; *WBRT =* whole brain radiotherapy; *CT =* chemotherapy; *ICIs =* immune checkpoint inhibitors

ST within one month after receiving RT is defined as RT synchrotron ST, and ST after one month after receiving RT is defined as ST after RT. Among 50 patients in the combined therapy arm, 33 received RT synchrotron ST, and 17 patients received ST after RT.

### Survival analysis in the matched dataset

**Fig. 1:**
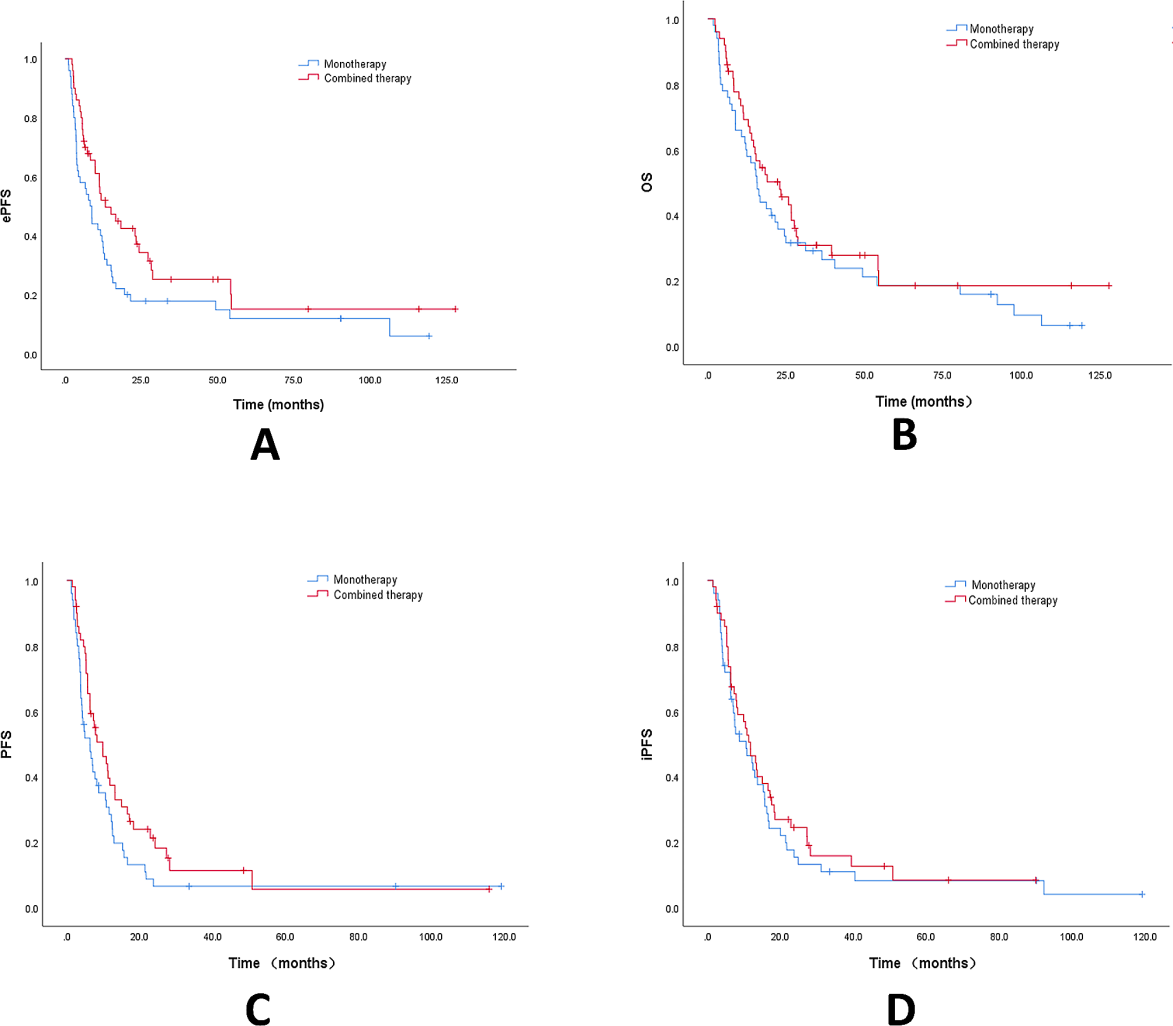
Kaplan–Meier survival curves of extracranial progression-free survival (ePFS) (**A**), overall survival (OS) (**B**), progression-free survival (PFS) (**C**), and intracranial progression-free survival (iPFS) (**D**) in the propensity score-matched dataset

The last follow-up was on September 1, 2023, with 31 patients still alive and 102 patients dead at the end of follow-up. After PSM, OS was analyzed in 50 patients in the monotherapy and 50 patients in the combined therapy (Fig. 1). The ePFS of the combined therapy was significantly longer than that of the monotherapy (*P* = 0.04), with a median ePFS of 13.2 months (95% CI, 6.6–19.8) in the combined therapy and 8.2 months (95% CI, 5.7–10.7) in the monotherapy. The median OS of the monotherapy and the combined therapy were 15.6 months (95% CI 12.3–19.0) and 22.8 months (95% CI 12.0-33.7), respectively, with a median PFS of 4.9 months (95% CI 2.3–7.5) and 9.8 months (95% CI 5.6–14.0). The median iPFS was 10.5 months (95% CI 4.5–16.6) and 11.7 months (95% CI 8.0-15.5), respectively. The differences in PFS (*P* = 0.057), OS (*P* = 0.309) and iPFS (*P* = 0.448) were not statistically significant.

Combined therapy (HR = 0.630, 95% CI 0.404–0.984, *P* = 0.042), performing PCI (HR = 0.369, 95% CI 0.160–0.849, *P* = 0.019), and with more than 5 BMs (HR = 2.077, 95% CI 1.324–3.258, *P* = 0.001) were significantly correlated with ePFS in univariate analysis (Table 3). In multivariate analysis, combination therapy was independently associated with better ePFS compared with monotherapy (HR = 0.617, 95% CI 0.394–0.964, *P* = 0.034); more than 5 brain metastases were associated with worse ePFS compared with 1–5 brain metastases (HR = 1.808, 95% CI 1.140–2.867, *P* = 0.012), and the effect of other variables on ePFS was not statistically significant (*P* > 0.05).

**Table 3:** Univariate and multivariate analyses of variables associated with extracranial progression-free survival

Variable	Univariate analysis				Multivariate analysis		
	HR	95% CI	*P*		HR	95% CI	*P*
Monotherapy v Combined therapy	0.630	0.404–0.984	0.042		0.617	0.394–0.964	0.034
Gender							
Female v Male	1.388	0.818–2.354	0.224				
Age, years	0.993	0.972–1.015	0.541				
KPS score							
90–100 v 70–80	1.015	0.970–1.061	0.532				
PCI							
No v yes	0.369	0.160–0.849	0.019		0.442	0.188–1.044	0.063
Number of brain metastasis							
1–5 v > 5	2.077	1.324–3.258	0.001		1.808	1.140–2.867	0.012

**Abbreviations:** *KPS =* karnofsky performance status; *PCI =* prophylactic cranial irradiation; *HR =* hazard ratio

The median BMFS was 7.9 months, and patients were divided into two groups: BMFS > 7.9 and BMFS≤7.9. The results of univariate analysis showed that there was no statistically significant effect of BMFS on iPFS (*P* = 0.255), ePFS (*P* = 0.677), PFS (*P* = 0.646), and OS (*P* = 0.728).

### Evaluation of treatment toxicities

The incidence of AEs is shown in Table 4. The rate of any grade AEs was 26% (13/50) in the monotherapy and 34% (17/50) in the combined therapy. The rate of grade 3–5 AEs was relatively higher in the combined therapy than in the monotherapy (12% vs. 4%, *P* = 0.269). However, the difference was not significant.

**Table 4:** Incidence of AEs

Treatmet-related AEs, n (%)	Monotherapy (*n* = 50)	Combined therapy (*n* = 50)	*P*
**Any grade**	13(26)	17(34)	0.383
Fatigue	6(12)	8(16)	
Nausea	8(16)	12(24)	
Diarrhea	0(0)	3(6)	
Neutrophil count decreased	5(10)	9(18)	
Leukocyte count decreased	6(12)	9(18)	
**Grade ≥ 3**	2(4)	6(12)	0.269
Neutrophil count decreased	2(4)	6(12)	

**Abbreviations**: *AEs =* adverse events

Only acute AEs was observed, and no chronic neurotoxicity such as cognitive impairment and memory impairment caused by therapy was observed.

## Discussion

This study retrospectively compared the survival outcomes of patients with simple BM after first-line treatment failure in LS-SCLC between the monotherapy and the combined therapy, with the aim of evaluating the clinical benefits of combined therapy. This study compared 50 patients in each group and adjusted for background factors related to clinical importance and prognosis through PSM. The main finding of our study is that compared to RT alone, RT combined with ST significantly prolongs the patient’s ePFS. The combination of treatment and less than 6 BMs significantly reduced the risk of extracranial recurrence in patients. BMFS is not associated with prognosis after BM. The rate of AEs was not significantly different between the two groups.

Currently, cranial RT is used as standard treatment for patients with SCLC BM. However, BM are a blood-borne disease, so RT as a local treatment may not be sufficient for systemic control of the disease. The combination of ST can reduce the occurrence of extracranial lesions, and the combination of systemic and local control can achieve overall survival benefits [11]. Therefore, many pilot studies conducted in cancer patients with BM have explored the efficacy of ST combined with RT. Several controlled studies have found that ST can improve the response rate to intracranial lesions, prolong patients’ PFS and even OS [14–17]. Koide et al. performed a retrospective study for patients with BM from the institutional disease database between 2016 and 2021. They evaluated the clinical benefits of ST combined with stereotactic radiosurgery (SRS) for BM. Their conclusion shows that the combined therapy group showed significantly longer PFS (median, 7.4 vs. 5.0 months, *P* < 0.001) and OS (median, 23.1 vs. 17.2 months, *P* = 0.036) than the monotherapy group [17]. However, The studies by Neuhaus et al. and Ge et al. had several notable differences from these studies, the results of their studies did not find a clinical benefit of ST [18, 19]. In most studies, patients often had metastases to extracerebral sites in addition to BM, and it is possible that, for ethical reasons, some studies were designed to allow ST to be administered to the RT alone group after completion of the treatment, resulting in non-comparable results for BM in the studies. To our knowledge, there are no reports comparing RT combined with ST with RT alone for simple BM after failure of first-line treatment for LS-SCLC. One of the advantages of this study is that the research subjects are patients with simple BM, and no patients in the RT only group receive ST, avoiding ethical issues. To further explore this issue, we conducted a retrospective study and collected 133 patients who met the inclusion criteria for the study.

The results of this study indicate that RT combined with ST can prolong ePFS in SCLC BM patients, without significant effects on OS, PFS, and iPFS. The proportion of SCLC BM patients receiving ST is not yet clear. However, in this study population, 50% of patients adopted a combination therapy regimen, indicating that combination therapy is not uncommon in clinical practice. A study conducted by European Organization for Research and Treatment of Cancer (EORTC) on the efficacy of WBRT for SCLC BM showed a higher recurrence rate after WBRT, proving that almost no patients have long-term benefits from WBRT. Therefore, even in SCLC patients with simple BM, it may be proposed to increase systemic therapy on the basis of WBRT [20]. The results of this study demonstrate that combined ST can reduce the occurrence of extracranial lesions and prolong the patient’s ePFS (*P* = 0.04). It may be due to the abundance of blood supply and lymphatic tissue in the lung tissue that cancer cells metastasis to the brain tissue mainly through lymphatic and blood circulation [21], and systemic therapy eliminates the tumor cells in the lymphatic and blood circulation, thus delaying the onset of extracranial progression. A study included 698 patients with SCLC BM, divided into four groups: the WBRT group (*n* = 178), the CT group (*n* = 129), the WBRT plus CT group (*n* = 273), and the best supportive care group (*n* = 118), and the results of the study demonstrated that WBRT plus CT improved the OS of patients with BM from SCLC, CT alone and WBRT alone did not show any survival benefits [22]. Although the number of patients is greater than that in this study, it includes patients with extracerebral metastasis other than BM, and the research results may be biased. In the present study, although there was no increase in OS and PFS in the combination therapy group, this is not surprising. It is possible that because of the small number of patients, we were unable to show the improved results when using combined therapy. ST is active, but it has been shown that the response of BM lesions to systemic CT (relative risk = 27%) is much lower than that of extracranial lesions (relative risk = 73%) [23, 24], and the drug diffuses poorly on the blood-brain barrier, resulting in limited impact of combined therapy on iPFS. Although the results of this study indicate that combined therapy does not reduce the risk of intracranial progression, we believe that cautious selection of ST may have the opportunity to improve iPFS. Studies have shown that ICIs with potential intracranial responses, such as atezolizumab and durvalumab, also have effects in the treatment of SCLC BM [25, 26]. A single-arm multicenter trial of platinum-etoposide plus atezolizumab for the treatment of untreated SCLC BM patients was recently initiated (NCT04610684). Therefore, RT combined with CT or ICIs is a promising research direction for SCLC BM [27].

In our study, multivariate regression analysis showed that combined therapy and fewer than 6 BMs significantly reduced the risk of extracranial recurrence in patients. Combined therapy was independently associated with better ePFS (HR = 0.617, *P* = 0.034), and more than 5 BMs were associated with worse ePFS (HR = 1.808, *P* = 0.012). PCI can not only eliminate small lesions that cannot be detected by imaging, but also increase the permeability of the blood-brain barrier, promote drug entry into the brain to eliminate lesions, reduce the occurrence of BM, and bring survival benefits to patients. A study has found that adding PCI to standard treatment for limited period SCLC reduces the 3-year incidence of BM from 59 to 33%, and improves the 3-year survival rate by 5.4% [28]. The incidence of BM in patients undergoing PCI decreased. The patients included in this study were those who developed BM after first-line treatment, so the number of patients who received PCI was relatively low (12%). History of PCI (*P* = 0.019) was significantly correlated with ePFS in univariate analysis, However, the results of the multivariate analysis showed that PCI was not associated with ePFS (*P* = 0.063). We believe that PCI, as a local treatment, has a limited impact on the risk of extracranial recurrence, which is consistent with the results of the multivariate analysis. In previous retrospective analysis, several prognostic factors have been identified for SCLC BM patients. Significant adverse prognostic factors include lower KPS, older age, presence of extracranial metastasis, and number of BMs [29, 30]. Due to the limited number of patients in previous analyses, SCLC BM patients were grouped with other solid tumors, especially non-small cell lung cancer (NSCLC), or the number of patients was too small to reasonably evaluate valuable prognostic factors [31, 32]. This study avoided the limitations of previous studies and conducted prognostic analysis on 100 patients with SCLC BM. We believe that it may be due to the fact that those with a higher number of BMs are prone to combine with more severe occupying effects and more brain tissue edema. In the absence of differences in other influencing factors, the tumor load may increase, leading to a shortened ePFS. Therefore, we believe that patients with more than 5 BMs should be actively treated with a combined therapy.

There is currently no consensus on whether the occurrence of BM is related to prognosis. The results of this study indicate that BMFS is not associated with prognosis after BM. The research results of Bernhardt on SCLC BM showed that patients with BMFS = 0–3 had better survival after BM than those with BMFS > 3 (*P* = 0.000) [31]. This study suggests that due to the high sensitivity of SCLC to early radiotherapy and chemotherapy regimens, but the early development of resistance to conventional treatment, the treatment effect of late onset BM is not satisfactory. The disadvantage of this study is that it did not consider the patient’s extracranial metastasis. To exclude the impact of extracranial metastasis on the results, this study selected SCLC patients with the first distant metastasis as BM. The results showed that BMFS had no significant impact on the survival of patients after BM occurred. This may be due to most patients have multiple BMs, which can cause significant damage to central nervous system function, and the general condition of patients can rapidly decline in a short period of time.

There are some limitations to this study. First, the results of this study may be confounded by other unobserved variables, and although we used rigorous statistical methods to adjust for baseline characteristics between the groups, these unobserved confounders may have been unbalanced between the groups and may have affected survival differences. Therefore, our findings should be interpreted with caution; second, the lack of data on the cause of death of patients (cancer-related or noncancer-related) and the fact that our analysis was not related to competing risks of death due to nonlung cancer may have led to bias in the calculated survival data. However, because the two groups did not show differences in age and KPS, we assumed that the competing risks of death were similar. Therefore, we believe that this bias, although present, did not significantly affect our results. A multicentre prospective study could be conducted in the future to further validate the conclusions of this study.

In summary, for patients with simple BM after first-line treatment of LS-SCLC, RT combined with ST shows the potential to improve ePFS compared to RT alone. Especially for patients with more than 5 BMs, an active combination therapy model should be adopted. The research results support the recent trend of combining systemic and local therapies, and encourage future randomized controlled trials to explore the best combination regimen and reasonable combination strategies.

## Data Availability

The datasets generated during and analyzed during the current study are available from the corresponding author on reasonable request.

## Abbreviations

RT: Radiotherapy
ST: Systemic therapy
BM: Brain metastasis
LS-SCLC: Limited-stage small cell lung cancer
ePFS: Extracranial progression-free survival
OS: Overall survival
PFS: Progression-free survival
iPFS: Intracranial progression-free survival
AEs: Adverse events
KPS: Karnofsky performance status
PCI: Prophylactic cranial irradiation
SRS: Stereotactic radiosurgery
WBRT: Whole brain radiotherapy
CT: Chemotherapy
ICIs: Immune checkpoint inhibitors

## References

[1] Siegel RL, Miller KD, Wagle NS, Jemal A. Cancer statistics, 2023. Cancer J Clin. 2023;73(1):17–48. 10.3322/caac.21763 36633525

[2] Petty WJ, Paz-Ares L. Emerging strategies for the treatment of small cell lung Cancer: a review. JAMA Oncol. 2023;9(3):419–29. 36520421 10.1001/jamaoncol.2022.5631

[3] Rossi A, Di Maio M, Chiodini P, Rudd RM, Okamoto H, Skarlos DV, et al. Carboplatin- or cisplatin-based chemotherapy in First-Line treatment of small-cell Lung Cancer: the COCIS Meta-Analysis of Individual Patient Data. J Clin Oncol. 2012;30(14):1692–8. 22473169 10.1200/JCO.2011.40.4905

[4] Lukas RV, Gondi V, Kamson DO, Kumthekar P, Salgia R. State-of-the-art considerations in small cell lung cancer brain metastases. Oncotarget. 2017;8(41):71223–33. 29050358 10.18632/oncotarget.19333 PMC5642633

[5] Kristjansen PE, Soelberg Sørensen P, Skov Hansen M, Hansen HH. Prospective evaluation of the effect on initial brain metastases from small cell lung cancer of platinum-etoposide based induction chemotherapy followed by an alternating multidrug regimen. Ann Oncol. 1993;4(7):579–83. 8395873 10.1093/oxfordjournals.annonc.a058592

[6] Lee JS, Murphy WK, Glisson BS, Dhingra HM, Holoye PY, Hong WK. Primary chemotherapy of Brain Metastasis in Small-Cell Lung Cancer. J Clin Oncol. 1989;7(7):916–22. 2544685 10.1200/JCO.1989.7.7.916

[7] Yomo S, Hayashi M. Is stereotactic radiosurgery a rational treatment option for brain metastases from small cell lung cancer? A retrospective analysis of 70 consecutive patients. BMC Cancer. 2015;15(1):95–102. 25879433 10.1186/s12885-015-1103-6 PMC4359776

[8] Putora PM, Fischer GF, Früh M, Califano R, Faivre-Finn C, Van Houtte P, et al. Treatment of brain metastases in small cell lung cancer: decision-making amongst a multidisciplinary panel of European experts. Radiother Oncol. 2020;149:84–8. 32445987 10.1016/j.radonc.2020.04.015

[9] Zhu Y, Cui Y, Zheng X, Zhao Y, Sun G. Small-cell lung cancer brain metastasis: from molecular mechanisms to diagnosis and treatment. Biochimica et Biophysica Acta (BBA) -. Mol Basis Disease. 2022;1868(12):166557. 10.1016/j.bbadis.2022.166557 36162624

[10] Rittberg R, Banerji S, Kim JO, Rathod S, Dawe DE. Treatment and Prevention of Brain metastases in Small Cell Lung Cancer. Am J Clin Oncol. 2021;44(12):629–38. 34628433 10.1097/COC.0000000000000867

[11] Li B, Dai ZX, Chen YD, Liu YW, Liu S, Gu XN, et al. Systemic therapy after Radiotherapy significantly reduces the risk of mortality of patients with 1–3 brain metastases: a retrospective study of 250 patients. Chin Med J. 2017;130(24):2916–21. 29237923 10.4103/0366-6999.220296 PMC5742918

[12] Tsui DCC, Camidge DR, Rusthoven CG. Managing Central Nervous System Spread of Lung Cancer: the state of the art. J Clin Oncol. 2022;40(6):642–60. 34985937 10.1200/JCO.21.01715

[13] Lin NU, Lee EQ, Aoyama H, Barani IJ, Barboriak DP, Baumert BG, et al. Response assessment criteria for brain metastases: proposal from the RANO group. Lancet Oncol. 2015;16(6):e270–8. 26065612 10.1016/S1470-2045(15)70057-4

[14] Gamboa-Vignolle C, Ferrari-Carballo T, Arrieta Ó, Mohar A. Whole-brain irradiation with concomitant daily fixed-dose Temozolomide for brain metastases treatment: a randomised phase II trial. Radiother Oncol. 2012;102(2):187–91. 22257825 10.1016/j.radonc.2011.12.004

[15] Zhuang H, Yuan Z, Wang J, Zhao L, Pang Q, Wang P. Phase II study of whole brain radiotherapy with or without erlotinib in patients with multiple brain metastases from lung adenocarcinoma. Drug Des Devel Ther. 2013;7:1179–86. 24133369 10.2147/DDDT.S53011 PMC3797237

[16] Liu Y, Liu XH, Wang Y, Zhu J, Xin Y, Niu K, et al. A study on different therapies and prognosis-related factors for 101 patients with SCLC and brain metastases. Cancer Biol Ther. 2017;18(9):670–5. 28812423 10.1080/15384047.2017.1360450 PMC5663412

[17] Koide Y, Nagai N, Miyauchi R, Kitagawa T, Aoyama T, Shimizu H, et al. Radiotherapy or systemic therapy versus combined therapy in patients with brain metastases: a propensity-score matched study. J Neurooncol. 2022;160(1):191–200. 36114369 10.1007/s11060-022-04132-2

[18] Neuhaus T, Ko Y, Muller RP, Grabenbauer GG, Hedde JP, Schueller H, et al. A phase III trial of topotecan and whole brain radiation therapy for patients with CNS-metastases due to lung cancer. Br J Cancer. 2009;100(2):291–7. 19127261 10.1038/sj.bjc.6604835 PMC2634726

[19] Ge XH, Lin Q, Ren XC, Liu YE, Chen XJ, Wang DY, et al. Phase II clinical trial of whole-brain irradiation plus three-dimensional conformal boost with concurrent topotecan for brain metastases from lung cancer. Radiat Oncol. 2013;8(1):238. 24125485 10.1186/1748-717X-8-238 PMC3853318

[20] Postmus PE, Haaxma-Reiche H, Gregor A, Groen HJ, Lewinski T, Scolard T, et al. Brain-only metastases of small cell lung cancer; efficacy of whole brain radiotherapy. An EORTC phase II study. Radiother Oncol. 1998;46(1):29–32. 9488124 10.1016/s0167-8140(97)00149-7

[21] Böttger F, Semenova EA, Song JY, Ferone G, van der Vliet J, Cozijnsen M, et al. Tumor heterogeneity underlies Differential Cisplatin Sensitivity in Mouse models of Small-Cell Lung Cancer. Cell Rep. 2019;27(11):3345–e33584. 31189116 10.1016/j.celrep.2019.05.057 PMC6581744

[22] Li H, Xue R, Yang X, Han S, Yang W, Song X, et al. Best supportive care Versus whole-brain irradiation, chemotherapy alone, or WBRT Plus Chemotherapy in patients with brain metastases from small-cell lung Cancer: a case-controlled analysis. Front Oncol. 2021;11:568568. 33732638 10.3389/fonc.2021.568568 PMC7957068

[23] Deeken JF, Löscher W. The blood-brain barrier and Cancer: transporters, treatment, and Trojan horses. Clin Cancer Res. 2007;13(6):1663–74. 17363519 10.1158/1078-0432.CCR-06-2854

[24] Seute T, Leffers P, Wilmink JT, ten Velde GP, Twijnstra A. Response of asymptomatic brain metastases from small-cell lung Cancer to systemic first-line chemotherapy. J Clin Oncol. 2006;24(13):2079–83. 16648509 10.1200/JCO.2005.03.2946

[25] Rittmeyer A, Barlesi F, Waterkamp D, Park K, Ciardiello F, von Pawel J, et al. Atezolizumab versus Docetaxel in patients with previously treated non-small-cell lung cancer (OAK): a phase 3, open-label, multicentre randomised controlled trial. Lancet. 2017;389(10066):255–65. 27979383 10.1016/S0140-6736(16)32517-X PMC6886121

[26] Paz-Ares L, Dvorkin M, Chen Y, Reinmuth N, Hotta K, Trukhin D, et al. Durvalumab plus platinum–etoposide versus platinum–etoposide in first-line treatment of extensive-stage small-cell lung cancer (CASPIAN): a randomised, controlled, open-label, phase 3 trial. Lancet. 2019;394(10212):1929–39. 31590988 10.1016/S0140-6736(19)32222-6

[27] Chen Y, Paz-Ares L, Reinmuth N, Garassino MC, Statsenko G, Hochmair MJ, et al. Impact of brain metastases on treatment patterns and outcomes with First-Line Durvalumab Plus Platinum-Etoposide in extensive-stage SCLC (CASPIAN): a brief report. JTO Clin Res Rep. 2022;3(6):100330. 35719865 10.1016/j.jtocrr.2022.100330 PMC9204731

[28] Aupérin A, Arriagada R, Pignon JP, Le Péchoux C, Gregor A, Stephens RJ, et al. Prophylactic cranial irradiation for patients with small-cell lung cancer in complete remission. Prophylactic cranial irradiation overview Collaborative Group. N Engl J Med. 1999;341(7):476–84. 10441603 10.1056/NEJM199908123410703

[29] Sperduto PW, Kased N, Roberge D, Xu Z, Shanley R, Luo X, et al. Summary Report on the graded Prognostic Assessment: an Accurate and Facile diagnosis-specific Tool to Estimate Survival for patients with brain metastases. J Clin Oncol. 2012;30(4):419–25. 22203767 10.1200/JCO.2011.38.0527 PMC3269967

[30] Sperduto PW, Chao ST, Sneed PK, Luo X, Suh J, Roberge D, et al. Diagnosis-specific prognostic factors, indexes, and treatment outcomes for patients with newly diagnosed brain metastases: a multi-institutional analysis of 4,259 patients. Int J Radiat Oncol Biol Phys. 2010;77(3):655–61. 19942357 10.1016/j.ijrobp.2009.08.025

[31] Bernhardt D, Adeberg S, Bozorgmehr F, Opfermann N, Hoerner-Rieber J, König L, et al. Outcome and prognostic factors in patients with brain metastases from small-cell lung cancer treated with whole brain radiotherapy. J Neuro Oncol. 2017;134(1):205–12. 28560661 10.1007/s11060-017-2510-0

[32] Gaspar L, Scott C, Rotman M, Asbell S, Phillips T, Wasserman T, et al. Recursive partitioning analysis (RPA) of prognostic factors in three Radiation Therapy Oncology Group (RTOG) brain metastases trials. Int J Radiat Oncol Biol Phys. 1997;37(4):745–51. 9128946 10.1016/s0360-3016(96)00619-0

