# Correction: The therapeutic effect of radiotherapy combined with systemic therapy compared to radiotherapy alone in patients with simple brain metastasis after first-line treatment of limited-stage small cell lung cancer: a retrospective study

**DOI:** 10.1186/s12957-024-03531-1

**Published:** 2024-09-17

**Authors:** Xinyu Gao, Tingting Liu, Min Fan, Hongfu Sun, Shixuan Zhou, Yuxin Zhou, Haolin Zhu, Ru Zhang, Zhanyuan Li, Wei Huang

**Affiliations:** 1 School of Clinical Medicine, Weifang Medical University, Weifang, China; 2 Department of Radiation Oncology, Shandong Cancer Hospital and Institute, Shandong First Medical University, Shandong Academy of Medical Sciences, Jinan, China; 3 Department of Nuclear Medicine, Shandong Cancer Hospital and Institute, Shandong First Medical University, Shandong Academy of Medical Sciences, Jinan, China

**Correction: World J Surg Onc 22, 89 (2024).**

10.1186/s12957-024-03372-y

Following publication of the original article [1], the author reported that there were typographical errors in the data in the following areas.

1. In the univariate analysis section of Table 3, the HR value “**0.063**” for Monotherapy vs. Combined therapy should be corrected to “**0.630**”.
2. In the “Survival analysis in the matched dataset” section of the Results, in the first sentence of paragraph 2, the phrase “Combined therapy (HR = **0.036**)” should be corrected to “Combined therapy (HR = **0.630**)”, and the phrase “performing PCI (HR = **0. 0.369**)” should be corrected to “performing PCI (HR = **0.369**)”.

The original article has been updated.
